# Paulownia Wood in the Production of Wood-Based Boards Used in Construction and Interior Design: Properties, Applications, and Prospects

**DOI:** 10.3390/ma19163384

**Published:** 2026-08-09

**Authors:** Dorota Dukarska, Mehr Unisa, Jakub Kawalerczyk

**Affiliations:** 1 Department of Mechanical Wood Technology, Faculty of Forestry and Wood Technology, Poznań University of Life Sciences, Wojska Polskiego 38/42, 60-627 Poznań, Poland; jakub.kawalerczyk@up.poznan.pl; 2 Independent Researcher, 80230 Joensuu, Finland; mehernisa000@gmail.com

**Keywords:** raw materials, particleboards, OSBs, plywood, solid wood panels, sandwich panels, environment

## Abstract

The growing demand for wood raw materials and the need to reduce pressure on forest resources are driving the search for alternative wood species for industrial applications. In this context, paulownia wood is a promising raw material for the production of wood-based materials due to its rapid growth, the possibility of harvesting it from plantations, and its favorable strength-to-weight ratio. Its use can contribute to the diversification of the wood industry’s raw material base. The aim of this article is to systematize the current state of knowledge regarding the potential use of paulownia wood in the production of boards intended for interior design and construction, including particleboards, oriented strand boards (OSBs), plywood, solid wood panels (SWPs), and sandwich panels. The article analyzed the availability and properties of paulownia as a raw material for the board industry, the advantages and limitations associated with its use in specific technologies, and the environmental aspects resulting from its utilization. It was found that paulownia wood can be a valuable raw material for the production of lightweight wood-based boards and sandwich panels. However, its effective use requires adjustments to production technology, particularly the pressing parameters, the gluing process, and the composition of the boards’ raw materials. Further research should focus on optimizing manufacturing processes and verifying the feasibility of implementing this raw material in industrial production.

## 1. Introduction

The development of the wood industry, and in particular the technology for producing wood-based boards, has for many years been linked to the search for new sources of wood raw materials. The use of materials should enable the production of materials with the required performance characteristics while simultaneously reducing production costs and increasing the efficiency of wood resource utilization. Issues related to the search for various types of alternative wood raw materials for the wood-based board industry, with particular emphasis on OSB and particleboard, have already been discussed in a previous paper by the authors Dukarska et al. [1]. It follows from this that recycled wood and fast-growing species, whose wood can serve as a substitute for raw materials traditionally used in board production, are of particular importance to the current wood industry. Among such species, paulownia (*Paulownia* spp.) is increasingly mentioned, and its plantation cultivation is expanding in many regions of the world. According to Nagata et al. [2] the earliest references to the use of paulownia date back several centuries B.C. Associated with both birth and death, the tree was held in high esteem and used for both medicinal and religious purposes. Paulownia trees have been widely cultivated since the 3rd century A.D. [3]. The current interest in this species stems both from its rapid growth, which allows it to reach large trunk sizes, and from the favorable timber quality achieved in just a few years of cultivation, enabling high biomass production of up to 50 t/(ha-year) [4,5]. In terms of its physical and mechanical properties, paulownia wood is most similar to willow and poplar wood [6]. It is characterized by low density, a favorable strength-to-weight ratio, good dimensional stability, and low thermal conductivity. These properties make it widely used, combining traditional craftsmanship with modern industrial technologies. For centuries, this raw material has been valued in Asia, where it is used to make furniture, veneers, plywood, and small everyday items such as kitchenware, spoons, rice boxes, and traditional footwear [6,7,8]. Paulownia wood is also used in musical instruments, where for centuries it has been a key material for making soundboards for traditional string instruments [3,9]. Although spruce (*Picea*) is typically used in the production of violins and pianos, paulownia exhibits better sound radiation quality (14.06 to 20.84 m^4^/kg·s) compared to spruce (12.41 m^4^/kg·s) [3]. These unique acoustic properties mean that in some instruments, paulownia replaces spruce, allowing for a new, distinctive tone [6,10]. Even today, paulownia wood is valued by manufacturers for crafting the cases of concert grand pianos as well as the bodies of electric guitars and basses [11]. It is worth noting, however, that due to the high content of tylos in its vessels, this wood has low porosity and poor sound absorption, which means it does not work as well as a sound-absorbing material [12,13].

Paulownia’s well-established role in traditional crafts now serves as a starting point for its use in modern technologies, ranging from environmental protection to advanced energy systems. Thanks to its high biomass production, this species is used in phytoremediation processes, effectively aiding in the removal of heavy metals such as copper, zinc, cadmium, chromium, nickel, and lead from the soil [12,14,15]. Paulownia, particularly the *P. tomentosa* species, is one of the most promising technologies for biological carbon capture and storage (CCS). This is because paulownia belongs to the C4 plant group, which is known for its exceptionally high capacity to photosynthetically fix carbon dioxide (CO_2_) compared to other plant species. They can convert CO_2_ into carbon-based products such as sugars, proteins, and lipids. Thus, they can serve as an effective tool in the fight against climate change, especially in agroforestry systems and in urban areas. It should be noted, that the ultimate effectiveness of CO_2_ capture by paulownia is strictly dependent on the plantation’s location, growing conditions, and management practices [16]. Magar et al. [17] demonstrated that sequestration efficiency also depends on the age of the trees and increases progressively as the plants age. For this reason, the CO_2_ sequestration values reported in the literature for paulownia vary significantly. According to Steier et al. [18], paulownia absorbs 100 tons of CO_2_/ha/year in Europe, and 200 tons of CO_2_/ha/yr in Morocco, while native trees absorb only 13–13.6 tons of CO_2_/ha/yr. Ghazzawy et al. [16], based on the literature and their own calculations, developed a model scenario, using which they estimated that a plantation of 1.5 million trees covering an area of approximately 2400 ha could sequester about 1.04 million metric tons of CO_2_ over a 10-year period. Careful selection of the best genetic traits from *Paulownia elongata S. Y. Hu × Paulownia fortunei (Seem.) Hemsl.* led to the development of a paulownia variety called Oxytree, which absorbs 10 times more CO_2_ than, for example, beech. It is estimated that paulownia absorbs 60–100 t/ha/yr, while its hybrids, such as Oxytree, can absorb as much as 111 t/ha/yr under the right conditions [19]. 

Other parts of the paulownia tree, such as the bark, roots, leaves, fruits, seeds, and flowers, which contain one or more bioactive compounds, are used in the pharmaceutical industry [13,20,21] and as valuable feed for farm animals [13,22,23]. Furthermore, in the energy sector, paulownia is a valued raw material for the production of renewable energy in the form of pellets and briquettes. According to Jakubowski [6], the calorific value of paulownia wood is similar to that of energy tree species already cultivated in Europe. For example, the paulownia hybrids 9501 and Shan Tong achieve gross heating values of 19.5 and 19.6 MJ/kg, respectively, which are comparable to those recorded for commonly used energy species such as willow (19.9 MJ/kg) and poplar (19.8 MJ/kg). The energy potential of paulownia wood also includes its use in pyrolysis and biomass gasification processes [24,25]. In addition, it can be used to produce liquid biofuels, including bioethanol and biohydrogen [4]. Up to 0.5 metric tons of ethanol can be produced from 1 metric ton of dry paulownia wood [26]. Its high cellulose content, approximately 47.85% [27], and fiber dimensions typical of hardwood species [28] make this wood an attractive raw material for the paper industry as well [29,30].

One of the most promising areas for the use of paulownia is the wood industry, where it is used in the production of construction and finishing materials, i.e., in the manufacture of lightweight particleboard, plywood, veneers, and blockboard, in which paulownia wood serves as the core layer [31,32,33]. Furthermore, modern technologies also enable the creation of advanced wood-polymer composites (WPCs) and biopolymers, and the wood’s susceptibility to densification and high-pressure treatment allows for improvements in its strength, hardness and durability [34,35,36], which opens up new avenues in the production of furniture and specialized construction boards. Despite the existing applications of paulownia in the wood industry, its potential in the production of board materials for construction and interior design has not yet been fully explored. 

In recent years, interest in the use of this type of raw material in the production of wood-based boards has increased, as reflected in the growing number of studies devoted to evaluating the physical and mechanical properties of the resulting materials. However, the available results are scattered and mainly concern selected types of boards, which makes it difficult to comprehensively assess the potential for using paulownia wood in the construction and interior design sectors. A review of the literature also indicates a lack of studies summarizing the available knowledge and comparing different types of wood-based boards in terms of their properties and potential applications. For this reason, it is therefore appropriate to systematize the available research results and conduct a comprehensive assessment of the potential of paulownia wood in the production of board materials intended for shaping functional spaces. 

Accordingly, the aim of this paper is to review the current state of knowledge regarding the use of paulownia wood as a raw material for the production of wood-based boards and to assess its potential for application in construction and interior design. It should be emphasized that this article extends the authors’ previous work on the use of alternative raw materials in the production of wood-based boards, focusing on the properties of paulownia wood and its potential applications in the technology of wood-based boards intended for various uses.

## 2. The Potential of Paulownia Wood as a Raw Material for the Wood-Based Board Industry

### 2.1. Scale of Paulownia Cultivation and Availability of Raw Material

To assess the potential of paulownia wood in the production of wood-based boards, it is necessary to characterize this raw material in terms of its availability, properties, and industrial applications. Previous analyses have shown a significant demand for traditional wood raw materials in the wood-based board industry [1], which justifies the search for alternative sources of wood. In recent years, paulownia has been attracting increasing interest, as reflected not only in the growing number of scientific publications on its properties and potential applications but also in the expansion of plantations. Paulownia belongs to the *Paulowniaceae* family, which consists of several species, including *Paulownia albiphloea Z.H.Zhu* (*P. albiphloea*), *Paulownia × australis T.Gong* (*P. australis*), *Paulownia catalpifolia T.Gong ex D.Y.Hong* (*P. catalpifolia*), *Paulownia elongata S.Y.Hu* (*P. elongata*), *Paulownia fargesii Franch (P. fargesii)*, *Paulownia fortunei (Seem.) Hemsl.* (*P. fortunei*), *Paulownia kawakamii T.Itô* (*P. kawakamii*), *Paulownia* × *taiwaniana T.W.Hu & H.J.Chang* (*P. taiwaniana*) and *Paulownia tomentosa (Thunb.) Steud.* (*P. tomentosa*). Among them, the most popular are *P. fortunei*, *P. tomentosa*, and *P. elongata*, and it is from these that numerous hybrids are most often created, which are intended to be resistant to local conditions [37,38].

**Figure 1:**
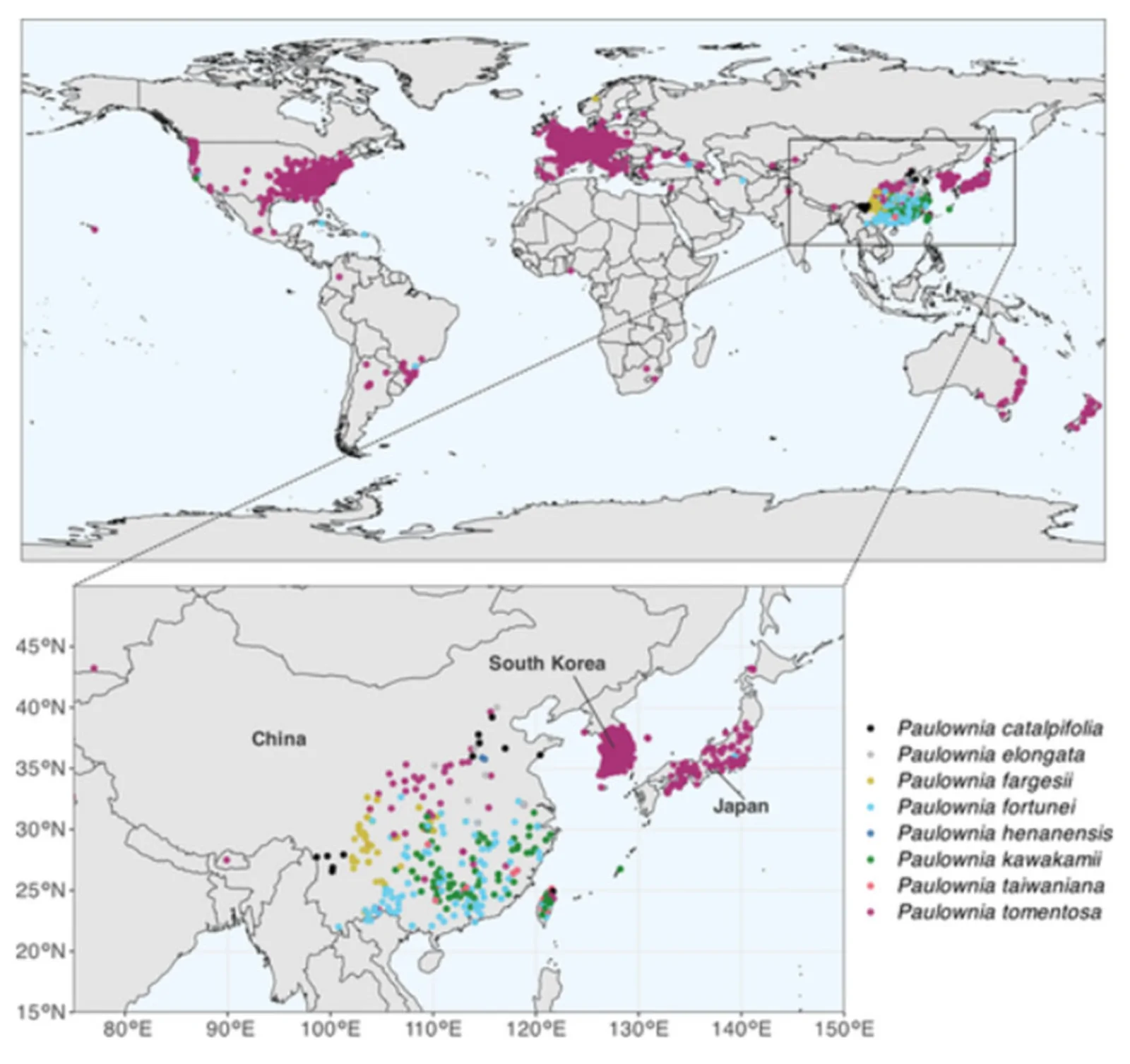
A map of the cultivation areas for the eight main species of paulownia, based on data from Global Biodiversity Information Facility (GBIF). Adapted from Young and Lundgren [45] (open access).

According to available estimates, the global area under paulownia cultivation is approximately 2–2.5 million ha (4–5 million ac). China holds a dominant position in the production of this type of wood, where paulownia is grown both on plantations and in agroforestry systems. Systems combining paulownia cultivation with agricultural crops cover approximately 1.3 million ha there [39]. Significant areas where this species is cultivated are also found in South Korea and Japan (Figure 1), although it is also gaining popularity in the U.S., Australia, and Europe [40,41]. From the perspective of using paulownia wood in the wood industry, cultivation efficiency is critical. This is because a single tree produces approximately 1 m^3^ of raw material over the course of 8–10 [42] or even, according to various sources, over the course of 5–7 years [43,44]. Under optimal conditions, the height growth of paulownia can exceed 6 m in a single growing season. This rapid growth rate is associated with the intensive development of the root system during the early stages of the tree’s growth, which ensures efficient uptake of water and nutrients, creating conditions for dynamic trunk growth and crown development.

**Figure 2:**
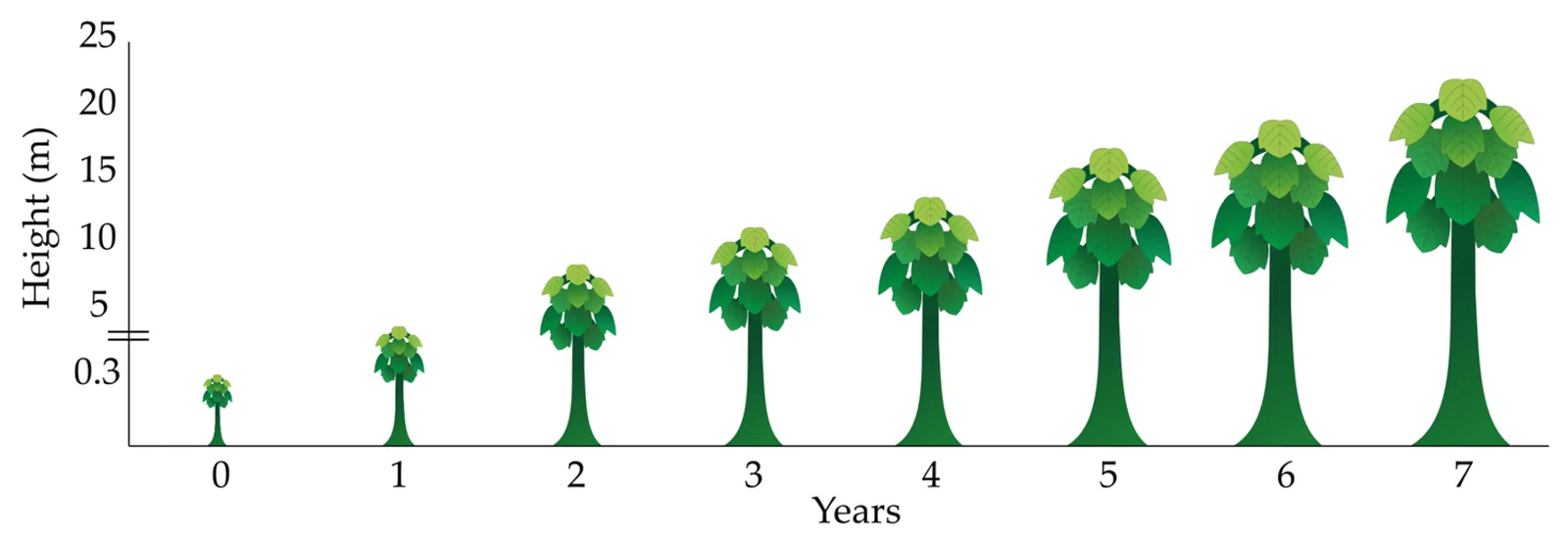
The growth of paulownia compared to other fast-growing trees [47].

Compared to conventional fast-growing tree species such as willow, poplar, eucalyptus, and red oak, paulownia exhibits significantly greater growth (Table 1, Figure 2) [44,46,47]. 

Another significant advantage of paulownia is its ability to regenerate through stem sprouts after timber harvesting. As a result, subsequent production cycles can be carried out without the need to replant the plantation, which increases the efficiency of raw material management and ensures greater supply flexibility compared to traditional forest species with significantly longer rotation periods [48,49]. 

**Figure 3:**
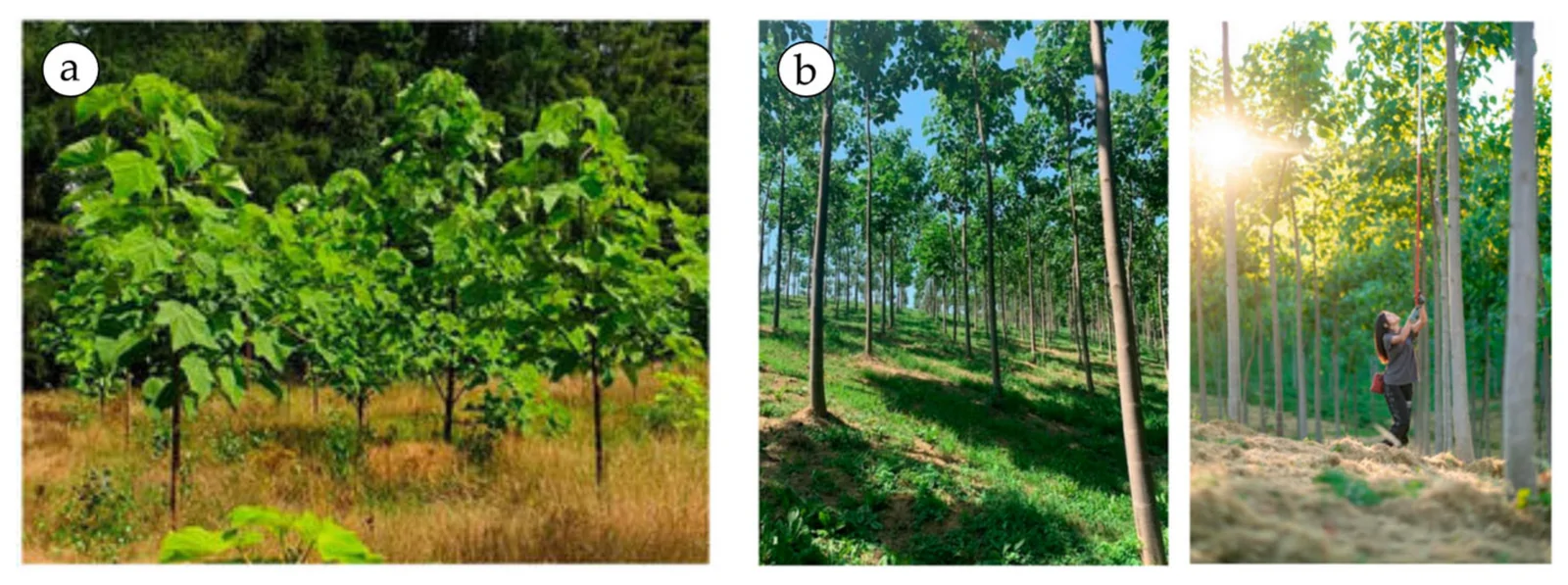
A photo of a paulownia plantation. Adapted from: (**a**)—Tomczak et al. [40], (**b**)—Barbu et al. [50] (open access).

Crop optimization is also crucial for the industry, including the selection of an appropriate planting density (Figure 3) and achieving high wood productivity per unit area. At a density of 2000 plants/ha, total production reaches approximately 330 t/ha. On the other hand, on plantations with a lower plant density, the production obtained may amount to approximately 150 t/ha [43].

### 2.2. Challenges and Limitations in the Cultivation and Use of Paulownia in the Wood Industry

Cultivation conditions play a key role in the proper development of paulownia crops and in determining the quality of the harvested wood. Of particular importance are climatic conditions, soil properties, the availability of water and nutrients, and the methods used to establish and manage plantations. Paulownia occurs naturally from tropical zones all the way to temperate climates. It can grow in temperatures ranging from −25 °C to 47 °C, although approx. 27 °C is considered optimal. Suitable conditions for growing paulownia exist at elevations of 700–800 m above sea level, and it is most commonly found at geographical latitudes between 40° N and 40° S [51,52,53]. It requires light intensities ranging from 20,000 to 30,000 lux. Thanks to their higher photosynthetic efficiency (based on C4-cycle enzymes) under the right conditions, paulownia trees are able to gain weight rapidly in a short period of time [6]. Furthermore, it grows on peat or sandy soils at an optimal pH range of 5.0–8.9 and a temperature of 15–16 °C [46,52]. Due to their ability to selectively uptake calcium (Ca^2+^) and magnesium (Mg^2+^) ions, they can also grow on saline soils (<1%) [51,52,53]. Research by Stefanov et al. [45] showed, however, that the *P. tomentosa* × *fortunei* hybrid is more tolerant to salinity than *P. elongata* × *elongata*.

**Figure 4:**
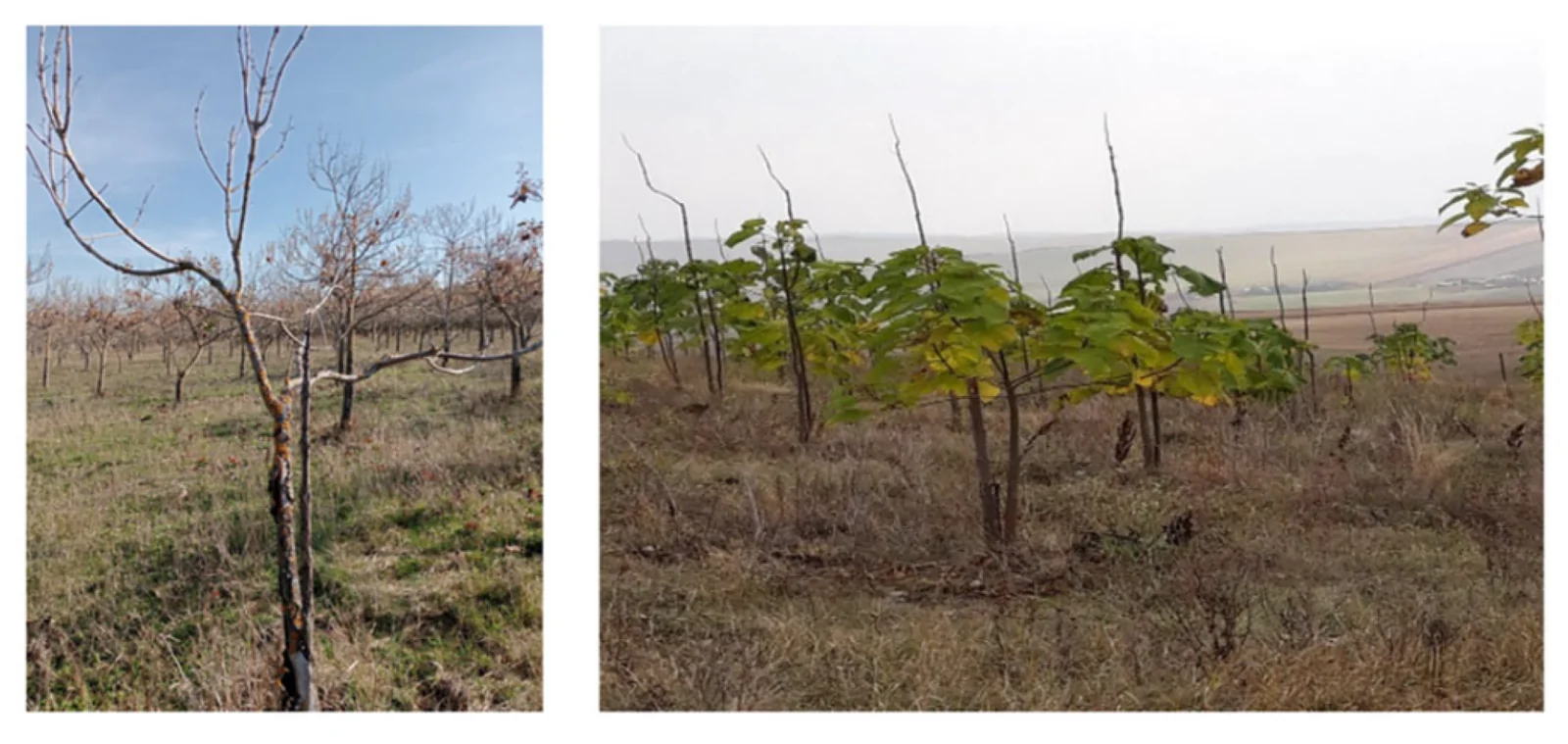
Paulownia plantation affected by winter frosts. Adapted from Negrușier et al. [54] (open access).

Despite its numerous advantages and high production potential, the wider use of paulownia wood in industry continues to meet a number of climatic, biological, technological, and market barriers that significantly affect the profitability and stability of production. A key limiting factor, particularly in regions with a temperate climate, is the trees’ sensitivity to extreme weather events, especially low temperatures (Figure 4). Species such as *P. tomentosa* show low tolerance to temperatures falling below −20 °C, while *P. fortunei* and *P. kawakamii*, and *P. fargesii*, which in regions such as Central and Eastern Europe can lead to damage to the tree stand, drastically reducing yields by up to 70% [6,54].

Another problem is the shorter growing season at these latitudes, which significantly limits biomass growth compared to the optimal conditions found in southern Europe or the Middle East. A major challenge is also the high water demand (up to 2000 L per seedling in the first year) [55], which, in the event of rainfall shortages, necessitates costly irrigation, as well as the susceptibility of young shoots to mechanical damage caused by strong winds [6]. Paulownia trees are also sensitive to soil salinity, although, as demonstrated by Stefanov et al. [56] the *P. tomentosa × fortunei* hybrid is more tolerant of salinity than *P. elongata* × *elongata*.

Biological and legal barriers are primarily related to the invasive nature of certain species, especially *P. tomentosa*, which, for example, has been designated in the United States as an alien species requiring strict monitoring or control [57]. However, in European countries, including the United Kingdom, Germany, France, Switzerland, Austria, Italy, and Spain, paulownia is not yet considered a conservation concern [58].

**Figure 5:**
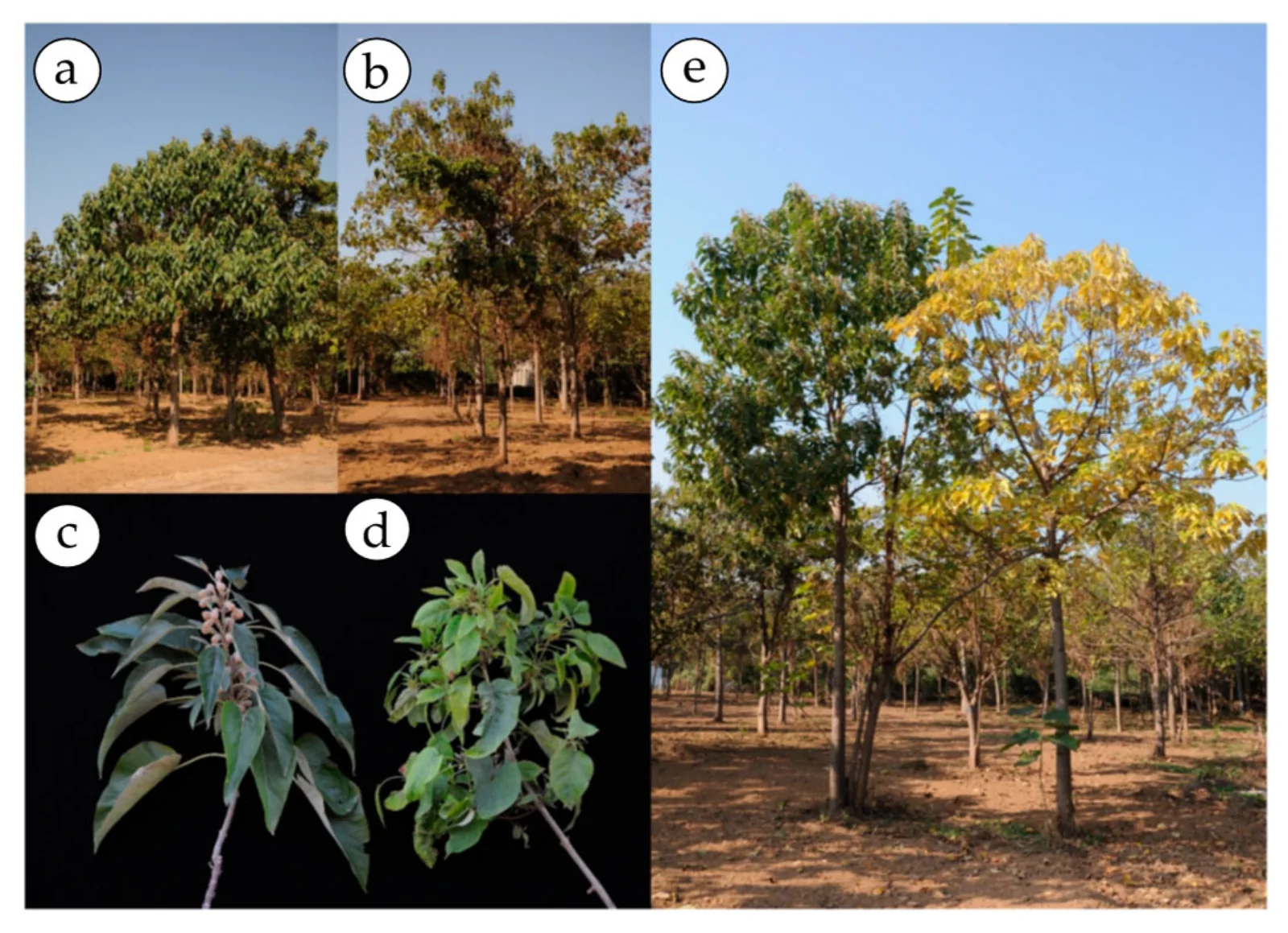
Typical symptoms of PaWB phytoplasma-infected paulownia trees: (**a**)—healthy tree, (**b**)—diseased tree, (**c**)—healthy branches, (**d**)—diseased branches, (**e**)—comparison of healthy tree (**left**) and diseased tree (**right**). Adapted from Zhang et al. [59] (open access).

Paulownia plantations are also susceptible to specific diseases, including Paulownia Witches’ Broom (PWB) disease, which is considered one of the most dangerous diseases affecting this species in East Asia and is caused by phytoplasmas (Figure 5) [59]. Soil pathogens of the genus *Phytophthora* are also significant; they cause root and root collar rot, which can lead to plant weakness and even dieback [58]. 

According to Negrușier et al. [54] the main factors limiting the expansion of paulownia cultivation are growers’ insufficient knowledge of agrotechnical practices and market conditions. From an economic standpoint, the availability of raw material is also limited by the high capital costs associated with purchasing in vitro seedlings and preparing the soil. In many countries, the development of the paulownia market remains limited, and the lack of a sufficiently developed infrastructure poses a significant barrier for producers, which may result in lower prices compared to international markets. Furthermore, the relatively long production cycle (6–15 years) increases investment risk. In the author’s opinion, the profitability of paulownia cultivation requires the simultaneous production of wood and biomass (a dual-purpose system), whereas plantations focused exclusively on the production of wood chips or biomass are characterized by very low, and sometimes even zero, profitability. All these factors mean that the success of paulownia plantations depends on the careful selection of varieties resistant to local climatic conditions, the application of appropriate agrotechnical practices, and understanding of market conditions, which in many cases are not sufficiently understood by growers. Planting density is another factor affecting the profitability of paulownia cultivation. For plantations intended for timber and biomass production, the most commonly recommended spacing is 4 × 4 m, 5 × 4 m, or 5 × 5 m, which corresponds to a planting density of 500–600 trees/ha. A higher number of trees, exceeding 800 trees/ha, is associated with an increase in investment costs of up to 20%, which can negatively impact the plantation’s profitability. Despite these limitations, paulownia is viewed as a promising alternative to traditional agricultural crops, allowing for the relatively rapid harvesting of timber with practical value.

A significant limitation to the wide use of paulownia wood in the wood industry is the fact that its availability varies greatly by region. As mentioned earlier, this raw material has played an important role in the wood industry of Asian countries for many years. In Europe, however, its use remains limited, mainly due to the small scale of cultivation and an underdeveloped market. Despite growing interest in this species, local supply chains and industrial applications for the raw material remain in the development stage in many countries.

### 2.3. Properties of Paulownia Wood

**Figure 6:**
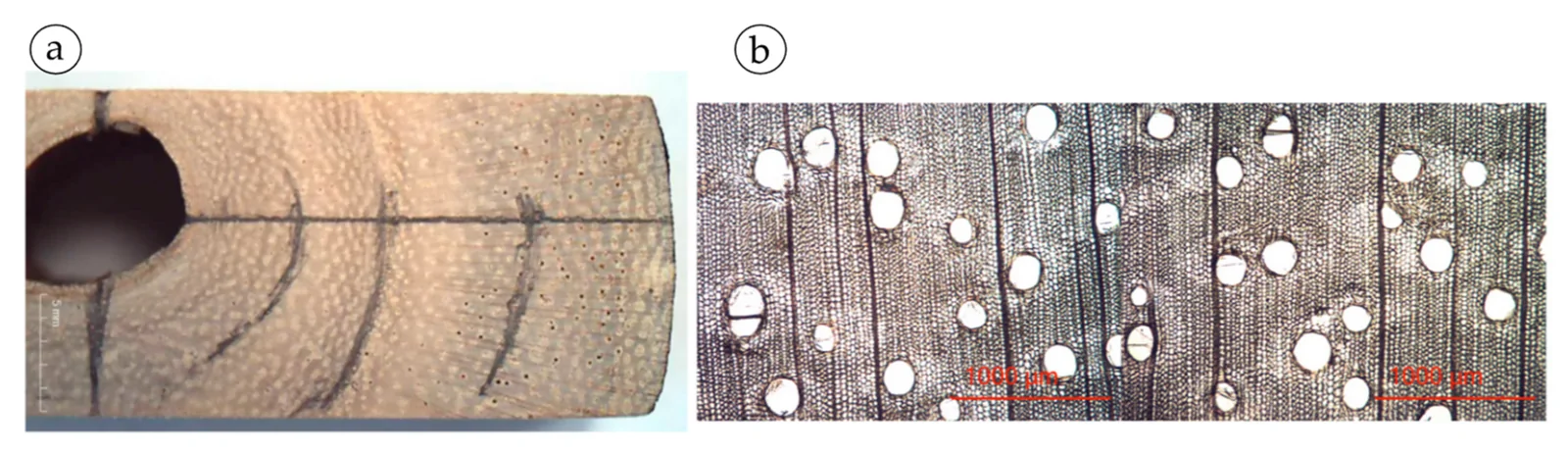
Cross-section of a young paulownia trunk: (**a**)—macroscopic structure, (**b**)—microscopic structure. Adapted from Tomczak et al. [40] (open access).

The potential uses of paulownia wood in the production of board materials are largely determined by its physical, mechanical, and anatomical properties. These characteristics influence both the processing of the raw material and the performance properties of the finished products, determining their suitability for specific applications. Paulownia wood is characterized by a light color, ranging from light yellow to light red, and a faint boundary between the sapwood and heartwood (Figure 6). The sapwood is usually narrow and consists of one or two annual rings. The boundaries of the annual growth rings are clearly visible in all cross-sections. The wood has a ring-porous or semi-ring-porous structure, depending on the growing conditions. The vessels are most often oval in shape and vary in size—the vessels of latewood are several times smaller than those of earlywood. They are surrounded by well-developed parenchyma, while the wood rays are mostly narrow and single-rowed and less often multi-rowed [6,10,60,61]. 

An analysis conducted by Doczekalska et al. [62] shows that the chemical composition of paulownia wood is as follows: 77.9% holocellulose, 43.3% cellulose, 34.7% hemicellulose, 25.5% lignin, and 11.5% extracts.

In terms of physical and mechanical properties, paulownia is similar to willows and poplars [10]. As noted by Lachowicz and Giedrowicz [38], in the literature, paulownia is considered the aluminum among trees due to the low density and stability of its wood. The density of paulownia wood is one of its most distinctive features and ranges from 220 to 350 kg/m^3^, most commonly around 270 kg/m^3^. The variation in these values stems primarily from differences among paulownia species and hybrids, the age of the trees, habitat conditions, and the moisture content of the wood at the time of measurement. Despite this variability, paulownia wood is classified among the lightest types of commercial timber, which is a consequence, among other things, of its highly porous structure. According to researchers, depending on the species, the porosity of paulownia ranges from 75 to 88%.

The low density of paulownia wood has a significant impact on its mechanical properties. The strength properties of this wood are generally lower than those of conventional species such as spruce, larch oak and even black poplar [63], although, as noted by Esteves et al. [64], its physical and mechanical properties increase with age, with the exception of the modulus of elasticity (MOE) and bending strength (MOR). However, it should be noted that an unambiguous assessment of the mechanical properties of paulownia wood is difficult due to the significant variability of the values reported in the literature. Strength parameters depend on many factors, including species or hybrid, tree age, site conditions, growth rate, and wood density. Consequently, the values for bending strength, modulus of elasticity, and compressive strength may vary significantly between individual studies, as indicated by the values presented in Table 2.

One of the important features of paulownia wood is its high dimensional stability, resulting from its low shrinkage and swelling. The tangential and radial shrinkage values are approximately 1.8–2.0% and 0.8–0.9%, respectively. The low anisotropy coefficient reflecting tangential vs. radial shrinkage (T/R) therefore plays a significant role in determining the dimensional stability of paulownia. Furthermore, the high porosity and, at the same time, low density of paulownia wood facilitate diffusion and the uniform distribution of moisture, as well as the dissipation of stresses, without causing cellular damage in the wood during moisture absorption due to swelling pressure [71]. This behavior of paulownia wood is also explained by the presence of narrower core rays, which form a single row up to 0.5 mm wide (multi-row rays may also occur). These rays constrain the wood in the radial direction and result in swelling values of up to 4%. The narrow width of the heart rays also has no effect on the higher swelling rate in the tangential direction [63]. In turn, Jo et al. [72] showed that the linear and volumetric shrinkage, as well as the coefficient of anisotropy, increased considerably after wood extraction. As a result, the wood has high resistance to deformation, such as warping, twisting, or cracking, even under varying humidity conditions [73]. Low total shrinkage values in the various anatomical directions reflect the wood’s low tendency to shrink in volume during the drying process. The result is minimal volume reduction between green and dry wood, favoring industrial yield and reducing losses during processing [69].

The properties of wood are largely determined by the conditions of the drying process, and selecting the appropriate conditions is crucial for its quality. For this reason, numerous studies have been conducted focusing on optimizing wood drying conditions. Taghiya et al. [74] and Tari et al. [75] and investigated the effect of various drying schedules on the properties of *P. fortunei*. They analyzed various schedules based on recommendations from the Forest Products Laboratory (FPL, mild—T6E3, moderate—T6E4, severe—T7E4) and based on diffusion schedules consisting of three initial moisture contents (MC) of 113% (Dif-1), 75.5% (Dif-2), and 53.5% (Dif-3). Based on this, they concluded that diffusion methods yielded the most favorable mechanical properties of the wood due to the beneficial effect of high temperature on increasing its plasticity. In contrast, FPL schedules with lower drying temperatures resulted in lower wood plasticity, which led to a decrease in its properties. In terms of gas and liquid permeability, which is crucial for subsequent impregnation and preservation of the wood, the mild FPL regimen designated as T6E3 was deemed the most effective. It provided the highest liquid permeability, the lowest warping, and the most uniform moisture profile.

An important aspect of assessing the suitability of wood for practical applications is also its fire resistance and biological durability. Paulownia wood exhibits exceptional fire resistance, which stems from its microstructure and chemical composition. This was explained by Li and Oda [76] who noted that the cell tissue of paulownia is very porous and similar to the structure of a honeycomb. Furthermore, this wood is characterized by the presence of large, isolated vessels, which limit the flow of oxygen into the interior of the material. Additionally, it contains a relatively small amount of lignin. As a result, when exposed to high temperatures, paulownia wood easily chars, releasing small amounts of flammable gases. The resulting char layer has lower thermal conductivity than the uncharred wood, thereby limiting further heat transfer into the interior of the material.

Paulownia is often considered a species that is also resistant to insects and fungi. According to Park et al. [77], *P. tomentosa* contains lignans, including paulownin, sesamin, and β-sitosterol, and is known to be resistant to insects and xylem-rotting fungi. Kirker et al. [78] demonstrated that *P. tomentosa* is resistant to brown rot and moderately resistant to white rot. They also observed that termites readily consumed extracted paulownia to a slightly higher degree than southern pine, though not significantly. In turn, Piętka et al. [79], while studying the paulownia variety ‘Cotevisa 2’, a hybrid of *P. elongata* and *P. fortunei* (COTE−2), under laboratory conditions, demonstrated that it is resistant to decay caused by the mycelium of *Heterobasidion annosum*. Despite the widespread recognition of paulownia as a durable species, there are, however, many reports of wood-decaying fungi found on paulownia. An example is the study by Paclt [80], which listed 25 fungal taxa from the class *Basidiomycetes found* on paulownia, including *Armillaria mellea*, which is responsible for root rot. Barton et al. [81] observed cases of paulownia dieback in New Zealand caused by a fungus of the genus *Armillaria*. Similarly, Milenković et al. [82] described a case of dieback affecting 80% of *P. tomentosa* trees on a 5-year-old plantation in Serbia, caused by the fungus *Trametes hirsuta*, which is responsible for white monilial rot.

## 3. Current State of Research on the Use of Paulownia Wood in Wood-Based Board Production

Research on the use of paulownia wood in the production of board materials includes an increasingly wide range of products that differ in structure, manufacturing technology, and intended use. In order to systematize the available knowledge, this section presents the results of research on specific groups of wood-based materials, with particular emphasis on their potential applications in the production of interior furnishings and in construction. 

### 3.1. Particleboards

Particleboard is one of the most commonly used wood-based materials, primarily due to its versatility, physical and mechanical properties suitable for a wide range of applications, cost-effectiveness, and the ability to enhance its surface using various techniques. According to FAOSTAT reports [83], global particleboard production in 2024 stood at 122 million m^3^. Approximately 68% of all particleboards produced were used in furniture manufacturing, 24% in construction, including the production of doors and flooring, and 1% in packaging production, while the remaining 7% were used in various other industrial sectors [84]. As a result, the particleboard industry faces the challenge of ensuring a stable and sustainable raw material supply. This has led to growing interest in the use of less conventional sources of wood, such as fast-growing species, including paulownia. Boards made from this type of wood can be used as furniture boards, structural boards, for the framing of skeleton constructions, and find application where boards with low weight but high insulation properties are required [85]. Research conducted to date in this area has focused primarily on three issues: (i) evaluating the potential for using paulownia wood as a primary or partial substitute for raw materials in board production, (ii) selecting appropriate material parameters for the raw material and the boards, and (iii) determining the optimal conditions for board production. 

Research on board materials made exclusively from paulownia wood appears to be of significant importance, as it allows for an assessment of this raw material’s true potential without the influence of other wood species. Among the earliest publications on this topic (according to the Web of Science database) is the 1980 paper by Clad and Pommer [85], in which the authors demonstrated that paulownia wood can be used to produce very lightweight particleboard without significant difficulty or the need for special production methods. The strength of such board is exceptionally high, even when the board’s density is below 450 kg/m^3^. 

A series of studies in this area was conducted by Nelis et al. [86,87,88]. The authors analyzed the possibility of substituting traditional wood particles with particles from *P. tomentosa* (kiri). For this purpose, single-layer particleboards were produced from 100% paulownia wood, bonded with UF resin, and had target densities of 350, 500, and 650 kg/m^3^. Additionally, for boards with a density of 350 kg/m^3^, mixtures of paulownia wood particles and industrial wood particles were used in ratios of 66:33 and 33:66. The tests showed that the paulownia-based boards had higher MOR and MOE values than the corresponding boards made from industrial wood particles. In this regard, these boards met the requirements for P1–P4 boards in accordance with EN 312 [89]. Equally satisfactory results were obtained for the internal bond (IB). These excellent properties of the boards may be due, among other things, to their denser structure, which is characterized by smaller and more evenly distributed air voids. This is likely a result of the high compressibility of lightweight paulownia wood during the pressing process. However, certain difficulties were observed with paulownia boards with a density of 650 kg/m^3^. It turned out that blisters appeared after the press was opened, which the authors attributed to the low bulk density of the paulownia particles, resulting in a voluminous mattress and low water vapor permeability of the mattress [86]. In a subsequent stage of the research, Nelis et al. [88] focused on analyzing the effect of wood density on the mechanical properties of the boards of three-layer particleboards with densities of 500 kg/m^3^ and 650 kg/m^3^. The experiments utilized combinations of beech wood particles, a high-density wood, and paulownia wood particles, using them interchangeably in the core and outer layers of the boards. It was demonstrated that the final properties of the boards are the result of an interaction between the density of the raw material used and its compaction level, which determines the effectiveness of the adhesive and the quality of the bond. In the case of lightweight boards with a density of 500 kg/m^3^, it was precisely the high degree of compaction of the paulownia particles that had a decisive impact on the boards’ strength, significantly outweighing the importance of the beech wood’s density. However, this relationship changed at a target board density of 650 kg/m^3^, where the density of the wood itself became the dominant factor shaping the boards’ properties, rather than the bonding efficiency resulting from compression. In the next study, Nelis et al. [87] demonstrated that the arrangement of paulownia particles is of significant importance in the structure of a three-layer boards. Their use in the outer layers of the boards (in a 50:50 ratio with industrial particles) significantly increased the MOR and MOE and had a positive effect on the dimensional stability of the boards by reducing their thickness swelling (TS) and water absorption (WA). In turn, the presence of paulownia in the core layer lead to a proportional increase in IB values, which, according to the authors, depend to a greater extent on the degree of particle compression and the resulting increased adhesion than on the density of the core layer itself. Furthermore, it was found that the surface layer had no effect on the screw withdrawal resistance, but it increased with the total amount of paulownia particles in the board.

**Figure 7:**
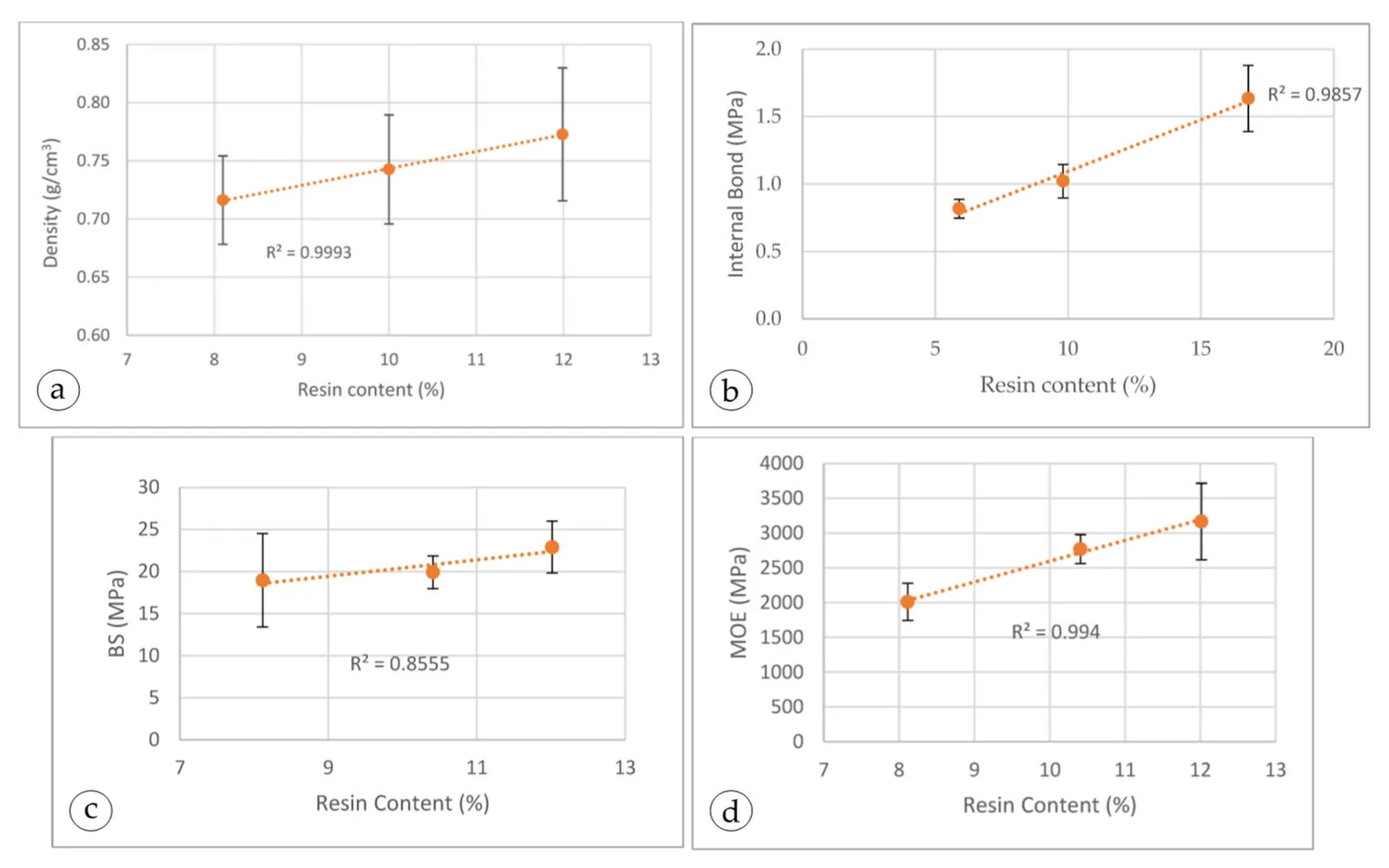
Selected properties of single-layer particleboard made from young age paulownia wood: (**a**)—correlation between board density and the size of paulownia wood particles; (**b**–**d**)—correlation between the degree of particle bonding and board strength. Adapted from Esteves et al. [90] (open access).

**Figure 8:**
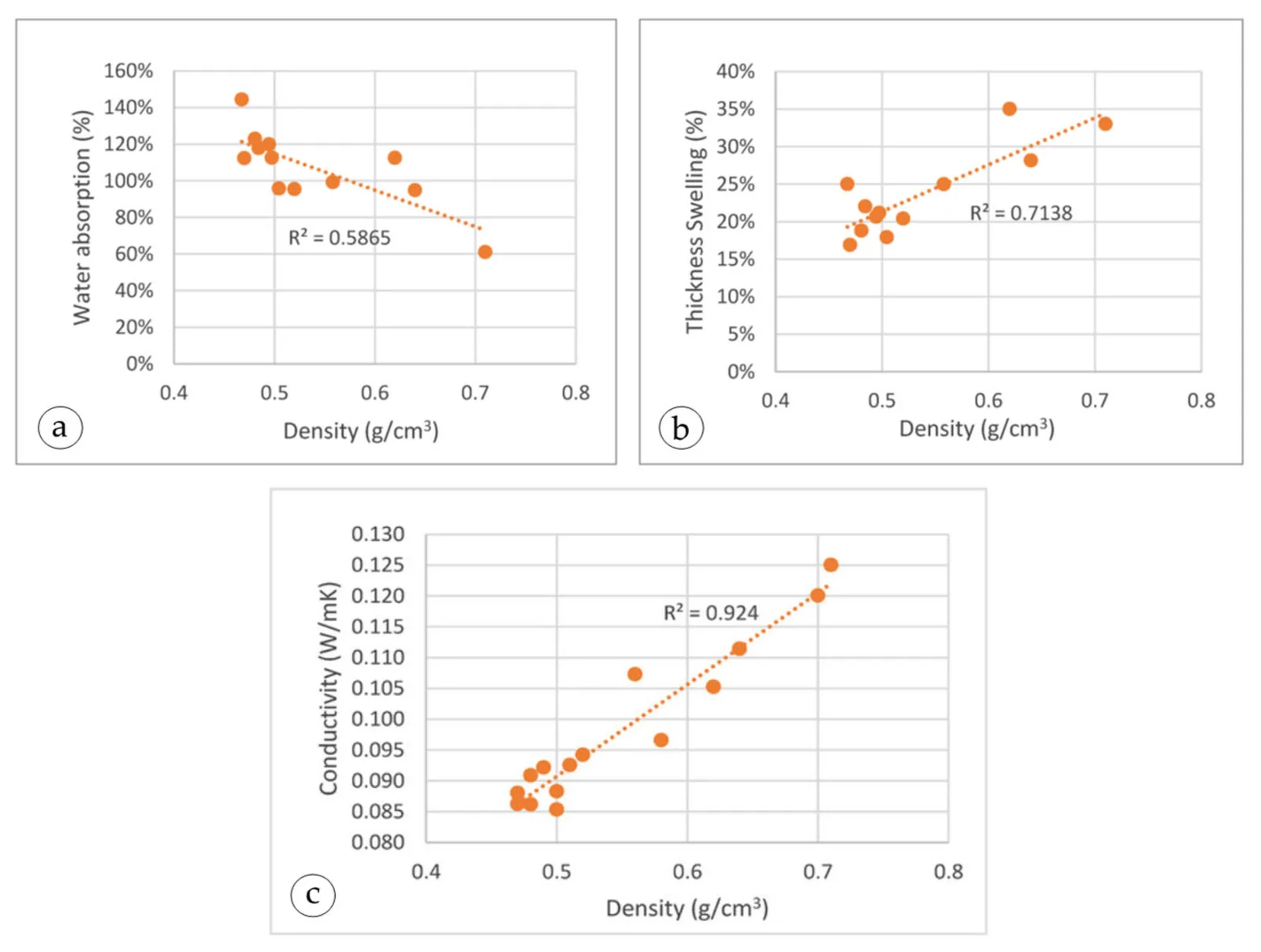
Effect of board density on their physical properties: (**a**)—water absorption, (**b**)—thickness swelling, (**c**)—thermal conductivity. Adapted from Esteves et al. [90] (open access).

A valuable supplement to these findings is the study by Esteves et al. [90], who used young age paulownia wood—only 3 years old—from plantations in Portugal to produce the boards. The authors produced single-layer particleboard using various degrees of UF resin gluing in the range of 8–12% and different particle sizes ranging from 0.25 to 0.4 mm, 0.4–1.18 mm, and >1.7 mm. Based on the tests conducted, it was determined that it was possible to produce boards from juvenile paulownia wood with densities ranging from 390 to 920 kg/m^3^, although to meet the standard requirements for P1–P2-type boards [68], these boards should have a density higher than 650 kg/m^3^. It was also found that larger paulownia particle sizes result in particleboards with lower density (Figure 7a), while a higher resin content leads to higher density. As expected, the increase in the degree of gluing and the higher density of the boards contributed to an improvement in their mechanical properties, i.e., MOR, MOE, and IB (Figure 7 b–d). The increase in board density, however, reduced WA while simultaneously increasing their TS and thermal conductivity (Figure 8).

Although increasing particle size may lead to a decrease in panel density and, consequently, in their strength, this relationship is not clearly defined. The results of Roellig’s et al. [91] research indicate that increasing particle thickness can have a beneficial effect on the strength properties of boards, leading to an increase in MOR, MOE, and IB values. These findings are also confirmed in the studies by Buresova et al. [41] and Alun et al. [92]. 

An important factor influencing the performance properties of particleboard is the pressing conditions used during the manufacturing process. This issue, given the potential to improve the performance properties of paulownia particleboard, was the subject of research by Rafighi and Tabarsa [93]. They concluded that increasing the pressing pressure from 30 to 40 bars and extending the pressing time from 8 to 12 min results in higher MOR (41.28 MPa) and MOE (4128 MPa) values. In the case of IB, the highest values (1.14 MPa) can be obtained using lower pressure and a shorter pressing time. With these pressing parameters, it is possible to produce paulownia wood boards with mechanical properties that exceed the requirements of the EN 312 standard [89]. This is confirmed by the results of the study by Kalaycioglu et al. [94], who also observed that an increase in board density and an extension of the pressing time contribute to an improvement in their strength parameters, enabling them to meet the requirements for boards intended for general applications. According to the authors, in addition to manufacturing parameters, the favorable properties of this type of board are also due to the higher lignin content and greater proportion of soluble substances in paulownia wood.

One of the most recent publications on particleboard and OSB manufactured from raw materials derived from fast-growing tree species is the study by Fauziyyah et al. [95]. The authors evaluated the performance characteristics of the boards depending on the wood species used and also analyzed their environmental impact through a screening life cycle assessment (SLCA). The results showed that boards made from fast-growing tree species exhibited a less uniform density profile than those made from coniferous wood. Furthermore, among the wood species studied—namely jabon (*Anthocephalus cadamba Roxb.*), albizia (*Paraserianthes falcatria L. Nielsen*), and paulownia (*P. tomentosa*)—paulownia and albizia wood showed great potential as raw materials for the production of particleboards and OSBs, particularly for use in dry and non-structural applications. This is indicated by the measured physical and mechanical properties of the board produced from these wood species. It was found that particleboards made from paulownia wood, glued with UF resin and having a density of approximately 525 kg/m^3^, exhibited lower TS and WA values after 2 h of water exposure than boards made from other fast-growing wood species, but higher than those made from spruce. After 48 h of soaking, the WA values for paulownia boards were comparable to those obtained for boards made from jabon and albizia wood. With regard to swelling, after prolonged exposure to water, paulownia boards exhibited values similar to those of albizia and spruce boards and lower than those of jabon. It should be emphasized, however, that the TS values for paulownia boards exceeded the standard requirements for P2-type boards, as did the boards made from other fast-growing species. In turn, the analysis of mechanical properties confirmed the very good performance of paulownia boards. For MOR, the highest average values were obtained among all tested variants. These values were statistically comparable to the results obtained for applewood boards and met the requirements for P4-type boards (according to EN 312 [89]). Similarly, the highest MOE value was obtained for paulownia wood boards, which met the P3 standard requirements in this respect. A trend similar to that observed for MOR was noted for IB. The paulownia boards achieved values comparable to those of jabon boards, while being significantly higher than those of albizia and spruce boards. Furthermore, they met the standard requirements for P4-type boards, which confirms their high application potential in particleboard production.

Given the topic of this article, which focuses on the use of paulownia wood in the production of boards for the construction industry, it is worth citing the paper by Chen [96], published in the 1990s, on gypsum boards containing paulownia. Although this research indicated the potential for using paulownia wood in the production of mineral-bonded boards, no further extensive studies exploring this topic were conducted in the subsequent decades. The author analyzed the properties of boards with a density of 1100 kg/m^3^ containing 30%, 25%, and 20% *Cryptomeria japonica*, *Paulownia × Taiwaniana*, and *Aleurites montana* (respectively), along with 70%, 75%, and 80% gypsum. The wood–gypsum board made from *Taiwanese paulownia* exhibited the highest MOR and MOE values, as well as screw holding strength comparable to that of *Aleurites montana*. However, it exhibited the highest TS and relatively high WA after 2 and 24 h of water immersion. The effect of gypsum content on the mechanical properties of the boards was not significant, but the TS and WA of the boards decreased as the gypsum content increased.

### 3.2. Strand Boards

In addition to particleboard, which is widely used primarily in the production of furniture, interior furnishings, and interior design products, paulownia wood is also being studied for use in the production of strand boards, which include oriented strand board (OSB), random strand board (RSB), and laminated strand lumber (LSL). Parallel-strand lumber (PSL) also belongs to this group; however, no reports on the use of paulownia wood for this purpose were found in the available literature. 

**Figure 9:**
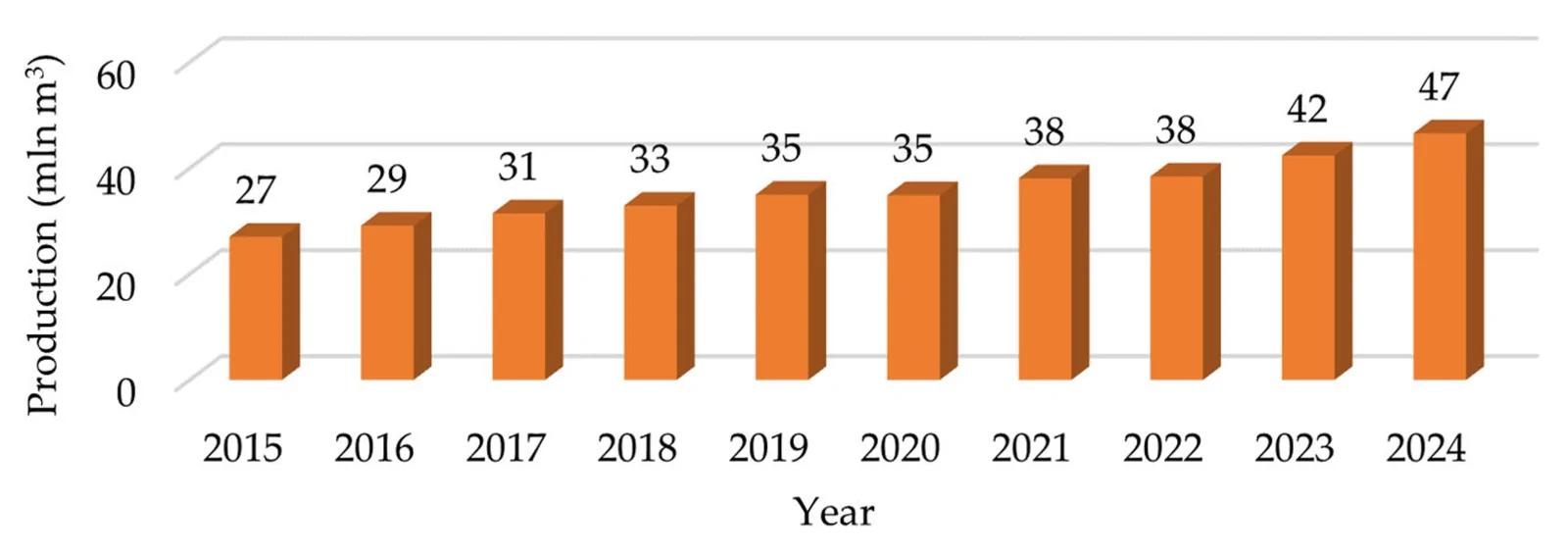
OSB production from 2015 to 2024, according to reports FAOSTAT [83].

Among these, OSBs are the most common. They constitute one of the most important groups of wood-based structural materials and are widely used in construction, including as wall, roof, and floor sheathing, as well as structural framing components. They are also used in the furniture industry, primarily for the production of upholstered furniture, bookcases, wardrobes, countertops, shelves, workbenches, and interior design elements, especially those with a loft or industrial style. The dynamic development of wood-based and prefabricated construction, as well as the growing use of OSBs in the furniture industry, have contributed to a significant increase in demand for these materials and, consequently, to an increase in their production in recent years (Figure 9).

Due to the wide range of applications, OSBs must meet high requirements regarding mechanical properties, dimensional stability, and moisture resistance. Therefore, assessing the suitability of paulownia wood for their production requires a detailed analysis of both the raw material’s properties and the technological parameters of the manufacturing process. In the study by Fauziyyah et al. [95] already mentioned in the previous subsection, demonstrated that paulownia wood has high potential as a raw material for OSB production, enabling the production of materials with properties comparable to or even better than those of OSBs manufactured from raw materials traditionally used in the industry. The OSBs produced as part of the study, glued with 4,4′-diphenylmethane diisocyanate (pMDI) adhesive and with a density of 518 kg/m^3^, although they did not meet the standard requirements for dimensional stability, as assessed based on swelling after 24 h of soaking in water, exhibited MOR values exceeding the requirements of EN 300 [97] specified for OSB/3 boards. Furthermore, the IB values met the standard requirements for OSB/2-type boards. In turn, Wang and Chen [98] investigated the effect of the proportion of paulownia wood strands on the mechanical and orthotropic properties of OSBs. In the context of OSBs, orthotropy refers to differences in mechanical properties (MOR and MOE) along the directions in which they are measured, i.e., in the longitudinal and cross directions and directions relative to the preferred orientation of wood strands [99]. The authors showed that boards containing up to 50% paulownia wood, in combination with Japanese cedar or Chinese fir, exhibited favorable MOR and MOE values. Ultrasonic analysis revealed a high degree of strand orientation in the outer layers of the boards, ranging from 93 to 97%, while the orthotropy index (V_0_/V_90_) ranged from 2.4 to 3.2. The results obtained demonstrate the high quality of the manufactured boards and confirm the potential of paulownia wood as a raw material for the production of wood-based structural materials.

The strength of paulownia wood OSBs is also influenced by modifications to the adhesive. This was confirmed by Salari et al. [100,101], who added nano-clay (organo-modified montmorillonite (MMT)) and nano-SiO_2_ to the UF resin. The addition of 3–5% nano-clay resulted in an increase in the boards’ MOR, MOE, IB, and screw and nail withdrawal strength. It also contributed to improved water resistance of the boards, as evidenced by a reduction in TS and WA. The boards’ hygienic properties were also improved, as demonstrated by reduced formaldehyde emissions. Pressing time also has a significant effect on the parameters of the boards. For boards modified with nano-clay, the optimal pressing time was 7 min; extending this time led to a deterioration in most physical and mechanical parameters. Similar results were obtained when nano-SiO_2_ was used. Its addition in amounts up to 3 phc resulted in improved strength properties, increased dimensional stability, and reduced formaldehyde emissions. However, in contrast to nano-clay, extending the pressing time from 7 to 10 min proved beneficial in this case. According to the authors, the nanoparticles used improve the mechanical properties of the panels by increasing the strength of the adhesive bond. This is due to their large specific surface area and high reactivity, which promote the formation of stronger interactions with the resin and improve its adhesion to the wood particles. At the same time, nanoparticles such as nano-clays or nano-SiO_2_ reduce formaldehyde emissions thanks to their barrier properties and their ability to adsorb and bind free formaldehyde, which improves the hygienic properties of the resulting boards. The boards obtained using both nanomaterials met the requirements of the EN 300 [97] for OSB/1 boards in terms of strength, but the TS values exceeded the standard requirements and were more than 25%. These observations were also confirmed in subsequent studies conducted by Bayatkashkoli and Faegh [102], who improved the properties of both OSB and LSL boards by using nano-clay, reporting these improvements at an addition level of 5–7%.

### 3.3. Plywood and Laminated Veneer Lumber

**Figure 10:**
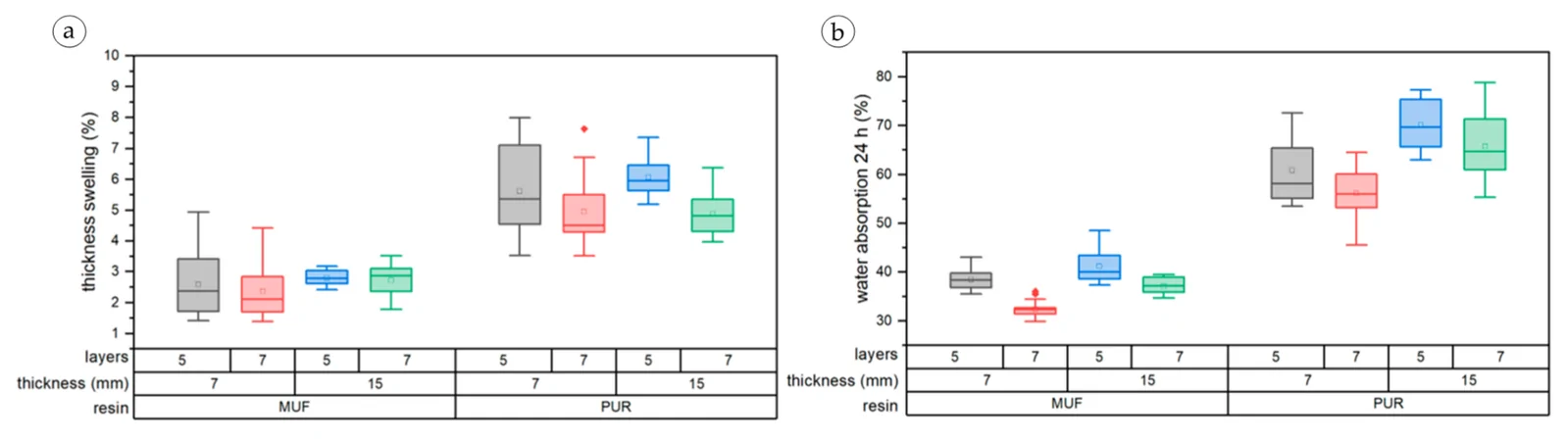
Thickness swelling and water absorption of paulownia-based plywood depending on the bonding agent, the number of veneer layers, and thickness: (**a**)—thickness swelling, (**b**)—water absorption. Adapted from Barbu et al. [103] (open access).

Plywood is one of the most extensively studied materials produced from paulownia wood. Interest in this application stems primarily from the wood’s low density, good machinability, and the ability to produce veneers of suitable quality. These characteristics make paulownia a promising raw material for the production of lightweight plywood. According to Barbu et al. [103] it represents a favorable alternative to balsa wood and in engineered wood products, offering a lighter and more cost-effective solution with the added benefit of a reduced environmental impact. The authors investigated the properties of plywood made from veneers sourced from *P. tomentosa × elongata* bonded with polyurethane (PUR) and melamine–urea–formaldehyde (MUF) adhesives. Based on this, they concluded that plywood with thicknesses of 7 and 15 mm and a low density ranging from 247 to 385 kg/m^3^ exhibits TS values between 2.47% and 5.34% and WA values between 35% and 68% (Figure 10). 

**Figure 11:**
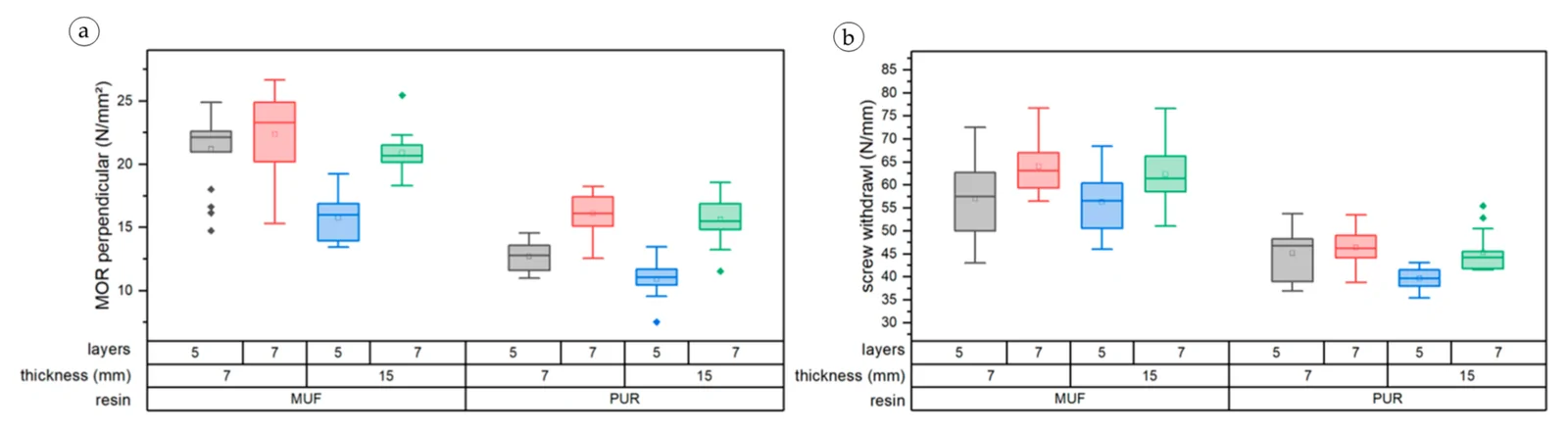
Bending strength in perpendicular direction and screw withdrawal resistance of paulownia-based plywood depending on the bonding agent, the number of veneer layers, and thickness: (**a**)—bending strength, (**b**)—screw withdrawal resistance. Adapted from Barbu et al. [103] (open access).

Their bending strength parallel to the fibers ranged from 22 to 35.8 N/mm^2^, and perpendicular to the fibers from 13.4 to 21.8 N/mm^2^. The modulus of elasticity ranged from 2824 to 3799 N/mm^2^ and from 1183 to 1825 N/mm^2^, respectively, while the shear strength ranged from 1.76 to 2.52 N/mm^2^ and the screw withdrawal resistance ranged from 41.9 to 60.6 N/mm. The authors also noted that increasing the number of layers improves the bending strength and screw withdrawal resistance of the plywood, which was attributed to the greater number of layers of relatively high-density adhesive (Figure 11). 

Despite its significantly lower density, paulownia plywood achieved bending strength values comparable to, and in some cases even higher than, those of poplar plywood used in lightweight structures. These results indicate that the combination of low density with favorable mechanical properties makes paulownia plywood a promising material for use in lightweight structures and a potential alternative to other materials used in such applications. The research by Barbu et al. [103] therefore highlight the cost-effectiveness and importance of paulownia plywood for the furniture industry.

Another important application of paulownia wood is the production of flexible plywood. The combination of low weight, adequate strength, and bendability makes these materials suitable for components that require bending and forming. For this reason, they are used, among other applications, in the furniture industry, interior finishing, and in structures with non-standard shapes. Sedighizadeh et al. [104] investigated the possibility of producing flexible plywood from a combination of poplar (*P. deltoides*) and paulownia (*P. fortunei*) veneers with thicknesses of 1 and 2 mm. The tests revealed that paulownia plywood, made with a 1 mm thick poplar veneer in the core layer, PVAc adhesive, and a veneer softener called Pro-Glue, was able to bend around a cylinder with a diameter of 6.5 mm, achieving a minimum radius of curvature without any signs of damage. The authors concluded that, depending on the intended application, poplar and paulownia can be used to produce flexible plywood. This is also confirmed by Nocetti et al. [5], who demonstrated that plywood made exclusively from paulownia wood may exhibit a greater number of surface defects, such as cracks, blisters, and areas of adhesive bleed, compared to poplar plywood and hybrid boards made from poplar and paulownia veneers. Furthermore, they had lower density, as well as lower strength and stiffness. Based on the results obtained, it was concluded that paulownia wood can be used for plywood production; however, the best results are achieved in hybrid structures where paulownia veneers are used as the inner layers, while the outer layers are made of poplar.

Such hybrid systems can also be used in the production of laminated veneer lumber (LVL), a new-generation structural material characterized by high strength and load-bearing capacity. Arabi et al. [105] analyzed the effect of various layer configurations of paulownia, poplar, and hornbeam veneers on the properties of a 7-layer LVL bonded with polyurethane adhesive. It was found that the use of hornbeam veneers in the LVL structure contributes to a significant increase in the density of this material and, consequently, its reinforcement. For example, in the case of plywood consisting of four layers of paulownia veneer and three layers of hornbeam veneer (in the outer layers and one middle layer), the MOR increased by 65% and the MOE by approximately 85%. However, it was observed that an increase in the proportion of hornbeam in the lamination resulted in increased thickness and transverse swelling of the LVL. The thickness and transverse swelling of solid hornbeam were higher compared to LVL made from poplar and paulownia wood. According to the authors, the alternating use of poplar and paulownia veneers with hornbeam is an effective method for improving the mechanical properties of LVL, while also offering potentially lower costs compared to technologies using fiber-reinforced polymers (FRPs). Despite the promising results of the proposed solution, the authors also attempted to improve the properties of paulownia-based LVL by using glass fibers (GFRPs) and carbon fiber-reinforced polymers (CFRPs) [106]. The research focused primarily on evaluating the effect of the type of reinforcing material on the mechanical properties and under water exposure. The study results showed that both solid wood and paulownia LVL had lower MOR and MOE values than those reinforced with CFRP, especially GFRP. The LVL manufacturing process itself led to an increase in material density due to the densification of the wood structure and adhesive penetration, while the use of GFRP or CFRP further intensified this effect. As the number of FRP layers increased, both MOR and MOE increased, with carbon fibers demonstrating greater reinforcement effectiveness. The authors demonstrated that the use of at least 3–4 layers of FRP enables the achievement of mechanical parameters that meet the requirements for structural materials, i.e., MOR of 50–80 MPa and MOE of 9000–12,000 MPa. Single-layer variants, despite significant improvements in mechanical properties, do not fully utilize the reinforcement potential due to premature cracking of the veneer layers. Increasing the number of reinforcing layers also changes the failure mechanism from brittle fracture to shear or compressive failure, which indicates a more favorable stress distribution across the element’s cross-section. The results indicate that multilayer FRP reinforcement, particularly with CFRP, is an effective method for enhancing the structural properties of LVL made from paulownia wood.

Ganji and Tabarsa [107] also proposed an interesting solution. They used a modified phenol-formaldehyde (PF) resin, in which a portion of the phenol (10% or 20%) was replaced with tannin, to produce paulownia plywood. The most favorable results were obtained with a resin composition of 20:80 (tannin), which yielded plywood with better performance properties compared to panels bonded with traditional PF resin. 

An alternative to conventional adhesive joints is the use of friction dowel welding technology. This method allows for the creation of joints without the use of additional bonding agents, utilizing the heat generated during the rapid insertion of the dowel into a prepared hole. Vitzthum et al. [108] investigated the possibility of welding wood dowels into low-density plywood made from poplar and paulownia wood without a pilot hole was investigated. Sharpened beech wood dowels with a diameter of 10 mm were inserted into plywood plates made from poplar and paulownia wood at a rotational rate of 1500 rpm with a manually operated drill press. The poplar samples showed very high values of withdrawal strength, reaching those of PVAc bonds. Connections with paulownia were also successfully achieved but with a weaker performance and more brittle failure.

### 3.4. Solid Wood Panels

**Figure 12:**
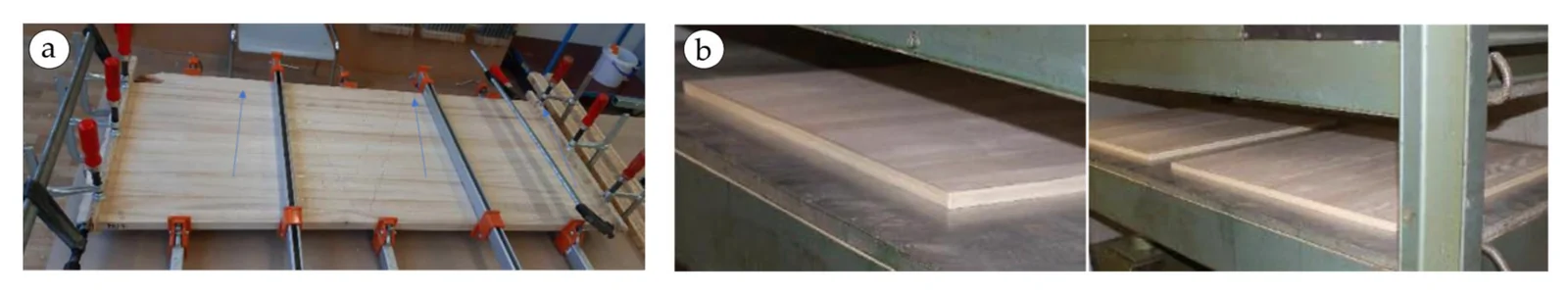
SWPs made of paulownia wood: (**a**)—single-layer, (**b**)—three-layer. Adapted from Barbu et al. [109] (open access).

Compared to particleboard or OSB, the use of paulownia wood in the production of laminated wood boards has so far been the subject of only a few studies. However, available literature data indicate that paulownia wood can also be used as a raw material in the production of solid wood panels (SWPs). Barbu et al. [109] analyzed the mechanical and physical properties of single- and three-layer SWPs with a thickness of 19 mm, depending on the type of adhesive, i.e., melamine–urea–formaldehyde resin (MUF), polyvinyl acetate (PVAc), and polyurethane (PUR), as well as the number of layers. In the case of single-layer edge-glued boards (Figure 12a), regardless of the adhesive used, no significant differences were found in their bending strength and stiffness, which were comparable to those of solid wood. The most favorable results were obtained for three-layer panels (Figure 12b) bonded with PUR adhesive, which also had the highest average density, at 283 kg/m^3^.

**Figure 13:**
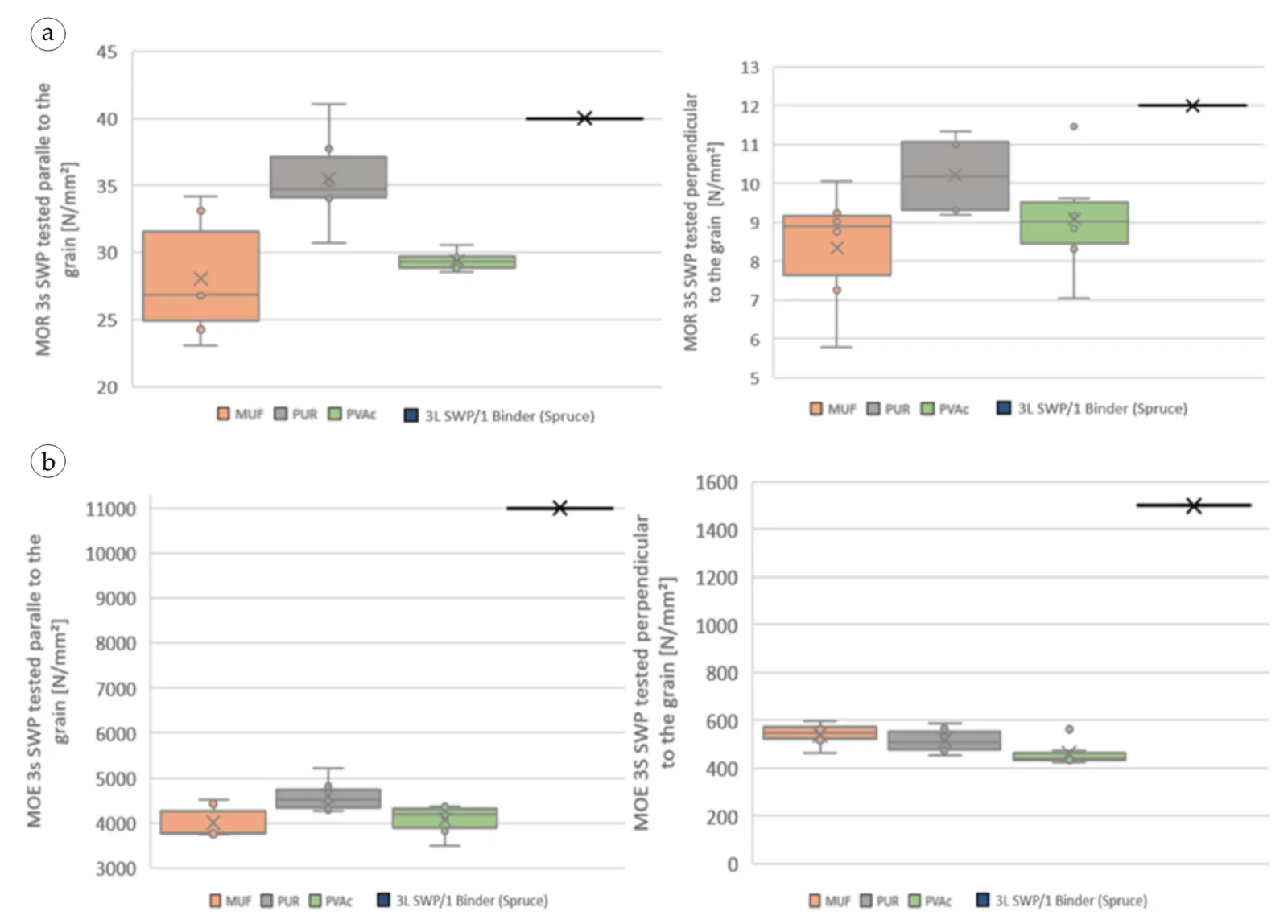
Bending strength (MOR) and modulus of elasticity (MOE) of the three-layered SWPs as a function of fiber orientation: (**a**)—bending strength, (**b**)—modulus of elasticity. Adapted from Barbu et al. [109] (open access).

Compared to boards bonded with MUF resin, they exhibited MOR values approximately 29% higher and MOE values approximately 15% higher. At the same time, the panels bonded with MUF resin had the lowest density, at approximately 277 kg/m^3^, which may have partially influenced the obtained mechanical properties. As expected, the highest values for both parameters were recorded in the direction parallel to the grain in the outer layers of the boards. Despite this, both MOR and MOE were nearly three times lower than the values obtained for the reference boards made of spruce wood (Figure 13).

Based on the results obtained, the authors concluded that SWPs made of paulownia wood could be used in lightweight structures, in the furniture and door industries, and in interior construction and transportation vehicles. It is worth noting that this conclusion is confirmed by industrial practice, as single-layer paulownia wood boards are already available on the market and are used in such applications [110]. Furthermore, researchers have noted that paulownia wood from European plantations resembles balsa wood and can effectively replace it in laminated structures as a core layer or in engineered products, such as three-layer solid wood panels. This allows for a reduction in the manufacturing costs of sandwich panels while simultaneously reducing the environmental footprint of the end product. 

### 3.5. Sandwich Panels

Sandwich structures are most commonly manufactured by combining thin, rigid face sheets with a thicker core, which increases material strength and stiffness while reducing its weight [111]. They constitute a well-studied group of materials whose properties and potential applications have been the subject of numerous studies. However, significantly less attention has been devoted to composite sandwich panels manufactured using lightweight wood species such as paulownia. Consequently, research in this area remains relatively scarce and represents a new, promising direction for the development of composite materials in which wood plays a significant role. Fiber-reinforced polymer (FRP) sandwich panels have attracted particular interest. Research by Sobhani et al. [112] confirms the high industrial potential of paulownia as a core material for sandwich panels, primarily due to its low density and favorable strength-to-weight ratio. In the analyzed systems, which used fiberglass facings bonded with polyester and epoxy resins, it was demonstrated that the thickness of the paulownia core has a significant impact on the density and technical parameters of the finished product. It was found that increasing the core thickness from 9 to 19 mm reduces the density of the entire panel, which results from a higher proportion of lightweight wood in the structure. This parameter is a significant factor influencing the fracture modulus, elastic modulus, and compressive strength of the panel. Regarding the lamination technology, it was found that epoxy resin provides better bonding than polyester resin, while the type of glass fiber used does not significantly affect the mechanical properties. The authors identified paulownia as a promising species for the production of lightweight and durable laminated panels, which represents a significant expansion of its applications beyond traditional particleboard. 

As part of their research on optimizing sandwich structures with a paulownia wood core, Wan et al. [113] A important technical solution turned out to be the creation of grooves on the paulownia surface, which were filled using the vacuum-assisted resin infusion method (VARIM). The cured resin inside the grooves formed a permanent mechanical bond, effectively anchoring the outer layers within the wood core structure. Analysis of samples with varying notch geometries (width, depth, and spacing) using the Double Cantilever Beam (DCB) method showed that the use of optimal notch geometry (i.e., 2.2 mm wide, a depth of 5.0 mm, and a spacing of 10 mm) increased the energy release coefficient to 1002 N/m, which represented a significant improvement compared to the 374 N/m observed in the reference specimens. The test results demonstrated that the presence of grooves significantly improved both the interphase properties and the machinability of the panels themselves. The results obtained have significant practical implications as they enable a wider use of paulownia in engineering structures.

The findings of Zhu et al. [114] indicate that paulownia, despite its low density, can be used in multi-story building structures, particularly in ceiling and floor elements that require adequate load-bearing capacity and stiffness. In the proposed solution, the core was reinforced with face sheets and internal webs made of glass fiber-reinforced polymer (GFRP), and the entire structure was manufactured using a vacuum-assisted resin infusion process. The results of four-point bending tests showed that important technical parameters, such as stiffness and ultimate bending strength, can be effectively optimized by modifying the panel’s geometry. The results showed that the bending stiffness and ultimate bending strength could be enhanced by increasing the web thickness, web height, and face sheet thickness, as well as by reducing web spacing. Taking this into account, it can be concluded that these panels represent a promising solution for floor systems in modern construction, combining the lightness of wood with the high load-bearing capacity of composites.

Yan et al. [111] investigated sandwich structures incorporating paulownia wood intended for use as wall panels. The researchers focused on analyzing the compressive behavior of GFRP-reinforced wall panels also manufactured using the VARIM. The effect of axial compression on panels of various heights was determined. The experimental results showed that the primary failure mode of these structures under compression was buckling. It was observed that the ultimate load-bearing capacities of the panel specimens depend closely on their geometry—these parameters improve as the panel’s height-to-thickness ratio decreases. Reducing this ratio from 51.5 to 38.6 resulted in an increase in the structure’s load-bearing capacity by as much as 124%. As with the floor systems analyzed earlier, the results of physical tests showed a high degree of agreement with the finite-element analysis (FEA), which allowed for the development of precise equations to predict the load-bearing capacity of wall panels with a lower height-to-thickness ratio. The authors also noted that this type of wall panel—which combines the advantages of lightweight, highly insulating wood with durable composite materials—exhibits greater load-bearing capacity than wooden structures. Therefore, they have the potential for use in construction, particularly in low-rise buildings.

Although this article focuses primarily on board materials, it is worth noting that paulownia wood has also been the subject of research on composite sandwich beams and columns. The results of this research confirm the potential for using this lightweight wood species not only in board materials but also in load-bearing structural elements. Wen [115] developed a cylindrical column made of fiberglass and paulownia wood using the vacuum resin infusion method. An analysis of its behavior under axial compression showed that increasing the thickness of the wooden core or facings lead to an increase in the column’s load-bearing capacity under axial loading. In the author’s opinion, the advantages of this type of column include low cost, rapid fabrication, product stability, light weight, high strength, and corrosion resistance, thanks to which it can be widely used not only for building columns but also for load-bearing columns for mine roads and coal mine roadways, and as part of the retaining structure for deep excavations. Qi et al. [116], while investigating the bending properties of sandwich beams with cores made of paulownia or southern pine, demonstrated that composite sandwich beams in flatwise bending tests failed under a lower load but exhibited greater deflection than those in sidewise bending tests. With an increase in the number of lattice webs, pseudo-ductility (gradual loss of load-bearing capacity after reaching the maximum load) was found to increase in the flatwise directions but decrease in the sidewise directions. Wan et al. [113] attempted to reinforce a composite column by filling holes drilled in the wood with glass fibers and resin. This solution had little effect on the bending strength but significantly improved the interlaminar properties of the structure. Subsequent researchers, Wang et al. [117], demonstrated that increasing the thickness of the GFRP skin and the density of the wood increases the ultimate axial load-carrying capacity of the columns. Other studies on sandwich columns using paulownia wood were also conducted by Yang et al. [118] and Kan et al. [119].

## 4. The Summary

A review of the available literature indicates that paulownia wood is an interesting raw material with potential for use in the production of wood-based boards for interior furnishings and even structural building components. Table 3 summarizes the main advantages, limitations, and directions for further development of paulownia wood applications in the production of specific types of boards. The appeal of paulownia as a raw material for the wood industry stems from its rapid growth rate, short rotation cycle, suitability for cultivation in temperate and subtropical climates, and favorable material properties, such as low density and a distinctive anatomical structure. Another significant advantage is the raw material’s plantation origin, which promotes supply stability and increases the predictability of the production process. At the same time, the potential for its wider use is limited by the restricted availability of the raw material resulting from the location of plantations, as well as climatic, biological, and market barriers. Paulownia wood exhibits a favorable strength-to-weight ratio, which makes it well-suited for use as a lightweight core in laminated structures and for the production of plywood. However, in the case of particleboards and OSBs, the wood’s low density leads to a lower degree of mat compaction during pressing, which may result in a deterioration of the boards’ mechanical properties. This effect can be mitigated by appropriately modifying the manufacturing process, including combining paulownia wood with higher-density raw materials, increasing the density and compaction level of the panels, optimizing the proportion and distribution of paulownia wood within the panel structure, selecting appropriate particle sizes, modifying the pressing parameters, and using a suitably selected binder or a modified version thereof.

Table 4, which presents a summary of the most important conclusions from the literature review, complements the previous overview covering the use of paulownia wood in various wood-based boards. In addition to technological issues, it also takes into account environmental aspects, which constitute a significant element in assessing the potential of this raw material.

In conclusion, it should be noted that the potential of paulownia wood in the production of wood-based boards (particularly those intended for construction) has not yet been fully exploited. Further research should focus on optimizing manufacturing technologies, particularly pressing parameters and gluing processes adapted to low-density wood, as well as evaluating the long-term physical and mechanical properties of the finished panels. Another promising direction is research on hybrid materials combining paulownia wood with higher-density species or other types of materials, as well as on modifying the surface and structure of the wood to improve its performance characteristics. Another important issue is the development of predictive models that account for the variability in the properties of plantation-grown wood, which could support its effective use in industrial settings.

It appears that further research and the development of technological guidelines and industrial standards will be crucial for fully realizing the potential of this raw material in the production of sustainable wood-based materials.

## Figures and Tables

**Table 1:** A comparison of paulownia growth with that of other fast-growing trees. Based on Janjić and Janjić [44].

Tree Species	Yearly Increase (m)	Height of a Tree Year Old Tree (m)	Maximum Height of Ripe Wood (m)
Paulownia (*Paulownia* spp.)	3–6	10.5–17.5	15–25
Hybrid willow (*Salix* spp. *hybrid*)	1.5–4	7.5–12	15–25
Black poplar (*Populus nigra*)	2.5–3.5	9–12	20–25
Hybrid poplar (*Populus deltoides*)	2.5–3.5	9–12	20–30
Texas red oak (*Quercus texana*)	2–2.5	7.5–9	15–20
Red eucalyptus (*Eucalyptus polyanthemos*)	2–2.5	6–9	10–15
Weeping willow (*Salix babylonica*)	1.5–2.5	4.5–9	15–20

**Table 2:** Comparison of selected properties of paulownia wood.

Species/Hybrid	Density (kg/m^3^)	MOR (MPa)	MOE (MPa)	Compression (MPa)	Shear (MPa)	Reference
*Paulownia tomentosa*	370	26.0	5200	22.0	-	[65]
	300	41.51	3492	22.14	7.03	[66]
*Paulownia fortunei*	325	47.25	5145	26.48	8.10	[67]
	370	40.4	3882	33.17	-	[68]
*Paulownia fortunei var. Mikado*		21.8	3129	14.2	3.80	[69]
*Paulownia tomentosa × elongata*	290	41.0	5000	23.0	-	[50]
*Paulownia COTE−2*	269	38.63	5153	-	-	[38]
*Paulownia Clone in vitro 112*	231	32.3	3800	19.9	4.10	[70]

**Table 3:** The use of paulownia wood in the production of wood-based boards–advantages, limitations, and directions for further research.

Type of Wood-Based Materials	Benefits of Using Paulownia Wood	Limitations on the Uses of Paulownia Wood	Directions for Optimization
Particleboards	the ability to reduce the density of the boards, high compressibility	lower density of the wood mat during pressing, reduced strength of the boards, high porosity, and high water absorption of the wood	the proper selection of the distribution and geometry of paulownia particles within the structure of the multilayer board, the adhesive or its modification, the pressing parameters, and combining it with other wood species
OSBs	lightweight structural boards	an appropriate degree of strand compression, high requirements for strength properties	increasing the strength of the boards
Plywood	good strength-to-weight ratio, good machinability, dimensional stability	low density, potential for surface defects (cracks, blisters, and areas where adhesive has seeped out)	hybrid systems using wood species with higher density and strength
SWPs	reduced component weight, good machinability, dimensional stability	the dependence of properties on the quality of adhesive bonds and the layer arrangement; limited board stiffness	multilayer and hybrid structures combining other wood species with higher strength and stiffness
Sandwich panels	lightweight, low-density core; favorable strength-to-weight ratio; dimensional stability; good energy absorption capacity; potential for reinforcement with composite materials; reduced panel manufacturing costs	the inherent strength and stiffness of the core under high loads, susceptibility to localized core crushing, limited strength of the face-core bond, risk of delamination if the layers are not properly bonded	increasing the strength of the core, improving interfacial properties, selecting appropriate face materials, developing new laminated composites, optimizing the gluing process

**Table 4:** Most important findings from the literature review on the use of paulownia wood in the production of wood-based boards for interior design and construction.

Area	Conclusion
Availability	plantation crops make it possible to plan raw material supplies and production
Properties of wood	low density, good machinability, rapid growth, favorable chemical composition and strength-to-weight ratio, compressibility, dimensional stability, low thermal conductivity
Potential applications	high, especially for lightweight wood-based materials and hybrid and composite systems
Limitations	crops grown mainly in Asian countries; climatic and market barriers; low density, which impairs the mechanical properties of the finished panels
Environment	rapid resource regeneration, short plantation rotation cycle, high CO_2_ absorption capacity, reduced pressure on natural forests, a renewable source of raw materials, potential for reducing the carbon footprint, suitability for cultivation on poor and degraded soils, phytoremediation capabilities, and the invasive nature of certain species

## Data Availability

No new data were created or analyzed in this study. Data sharing is not applicable to this article.
